# Surgical treatment of carpal tunnel syndrome in advanced-stage upper extremity lymphedema: A case report

**DOI:** 10.1097/MD.0000000000025872

**Published:** 2021-05-21

**Authors:** Soo-Byn Kim, Kyung-Chul Moon

**Affiliations:** Department of Plastic Surgery, Korea University College of Medicine, Seoul, South Korea.

**Keywords:** breast cancer, carpal tunnel syndrome, lymphedema, lymphovenous anastomosis

## Abstract

**Rationale:**

Despite significant advances in microsurgical techniques, simultaneous release of transverse carpal ligament (TCL) and lymphovenous anastomosis (LVA) surgeries may be effective for treatment of carpal tunnel syndrome (CTS) and advanced-stage lymphedema. This case report describes the successful treatment of lymphedema with LVA in a patient with CTS and advanced-stage lymphedema.

**Patient concerns:**

A 60-year-old female patient was referred to our lymphedema clinic with a 12-year history of chronic, acquired, right upper extremity lymphedema and CTS following right mastectomy and axillary lymph node dissection and adjuvant chemoradiotherapy for treating breast cancer.

**Diagnosis:**

According to the indocyanine green lymphography, magnetic resonance lymphangiography, and electromyography, the patient was diagnosed with CTS and advanced-stage lymphedema (International Society of Lymphology late stage 2).

**Intervention:**

Release of the TCL was performed first, followed by LVA at the wrist, forearm, and antecubital area. The right arm was compressed and elevated immediately postoperatively and postoperative compression bandage therapy with 35 to 40 mm Hg pressure was instituted following surgery.

**Outcomes:**

After 2 simultaneous surgeries, the patient had significant circumference and volume reduction of the right hand. The CTS and lymphedema symptoms have decreased following synchronous TCL release and LVA surgeries.

**Lessons:**

Simultaneous LVA and release of the TCL may be effective and safe in patients with advanced lymphedema and CTS.

## 1 Introduction

Breast cancer-related lymphedema is a chronic condition characterized by lymphatic vessel ectasia and valve dysfunction, followed by the reflux of lymphatic fluid into the interstitial space. Lymph stasis may lead to a chronic inflammatory process, resulting in adipose tissue differentiation and fibroadipose tissue deposition.^[1]^ Lymphedema is traditionally treated with decongestive physiotherapy and compression garments. Controversy exists regarding a cause–effect association between lymphedema and the development of peripheral neuropathies.^[2]^ However, increasing abnormal fibroadipose tissue deposition and pressure in upper extremity lymphedema patients and repetitive compression garment therapy may act as risk factors for the development of carpal tunnel syndrome (CTS), particularly in patients with advanced-stage lymphedema.^[3,4]^

Gunnoo et al^[5]^ demonstrated a significant increase in 5-month postoperative lymphedema volume compared with the preoperative volume in patients with lymphedema and CTS. Recent advances in supermicrosurgery have evolved to treat lymphedema surgically. Koshima et al^[6]^ made a key modification by introducing supermicrosurgical lymphovenous anastomosis (LVA), which uses lymphatics and venules <0.8 mm. Therefore, LVA may effectively improve lymphedema after release of transverse carpal ligament (TCL) in patients with CTS and advanced stage lymphedema.

However, no studies have reported simultaneous release of TCL and LVA surgeries for patients with CTS and advanced stage lymphedema. This case report describes the successful treatment of lymphedema with LVA in a patient with CTS and advanced-stage lymphedema.

## 2 Patient and methods

### 2.1 Patient and preoperative evaluation

A 60-year-old female patient was referred to our lymphedema clinic with a 12-year history of chronic, acquired, right upper extremity lymphedema in 2020. The patient has provided informed consent for publication of the case. The patient had a history of right breast cancer, which was treated with right mastectomy and axillary lymph node dissection and chemoradiation therapy in 2008, and a body mass index of 27.4 kg/m^2^. She had right upper extremity lymphedema (International Society of Lymphology late stage 2), and first noticed signs and symptoms of numbness and tingling sensation in her right hand affecting the thumb, index, and middle fingers since the symptoms of lymphedema worsened from 2012. She underwent electromyography and the nerve conduction study showed moderate CTS in both hands and that the right hand with lymphedema was worse than the unaffected hand. The results of the median sensory nerve conduction test from the middle finger to the wrist showed latencies of 4.6/5.6 and 4.9/5.9 ms, respectively. The bilateral median motor and sensory responses showed prolonged latencies and low amplitudes. In addition, the needle electromyography showed polyphasic motor unit action potentials with reduced recruitment patterns in the bilateral abductor pollicis brevis muscles. The patient also complained of severe right upper extremity heaviness, pain, infection, and intermittent sepsis that required hospitalization. She underwent medical treatments including decongestive physiotherapy for years, daily manual and mechanical lymphatic drainage, and wore compression garments. However, the lymphedema symptoms waxed and waned and were refractory to nonsurgical management.

**Figure 1:**
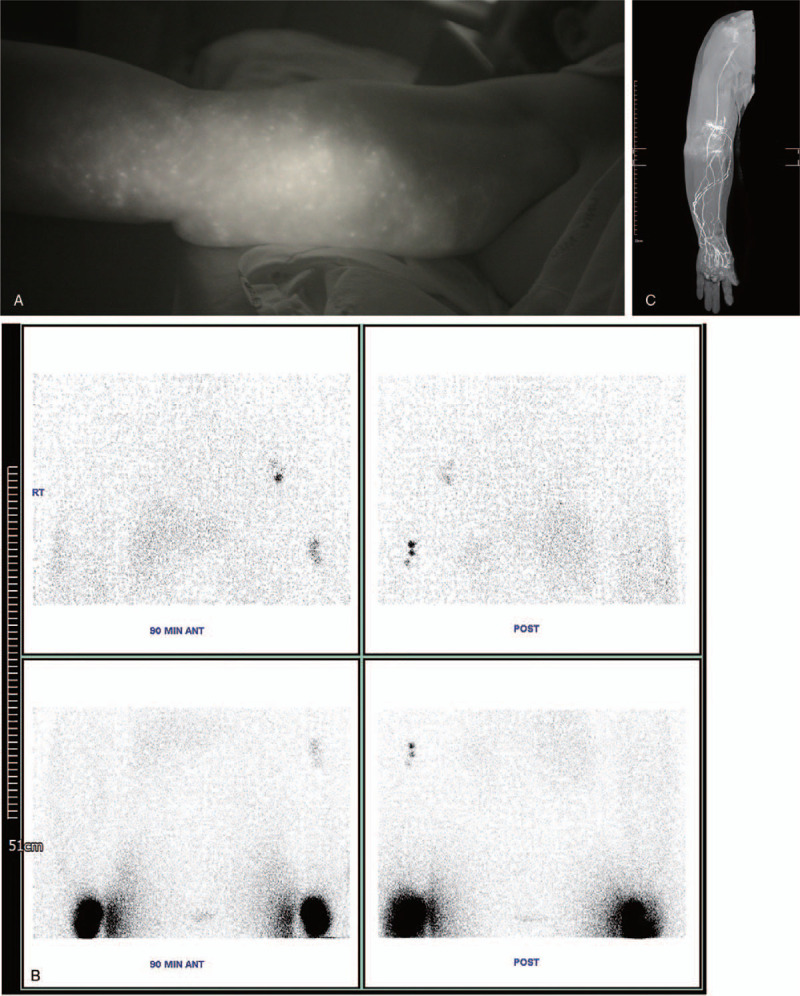
A patient with right upper extremity lymphedema and carpal tunnel syndrome. (A) Indocyanine green lymphography. (B) Lymphoscintigraphy. (C) Magnetic resonance lymphangiography. This patient had International Society of Lymphology late stage 2 lymphedema with confluent dermal backflow and patent lymphatic vessels were visualized.

A milliliter of indocyanine green (ICG) mixed with 2% lidocaine was injected subcutaneously at the second web space of the affected extremity at the bedside. After 2 hours, fluorescence images of lymphatic vessels were obtained with a near-infrared camera (Moment K; IANC&S, Seoul, Korea) and showed confluent dermal backflow in the forearm and few patent lymphatic vessels were visualized on the right upper extremity with severe lymphedema. Lymphoscintigraphy and magnetic resonance lymphangiography were performed before surgery and magnetic resonance lymphangiography identified functioning vessels in the wrist and forearm (Fig. 1). Therefore, we planned simultaneous release of TCL and LVA to treat the severe lymphedema that the patients experienced for >10 years. The circumference of the affected and unaffected upper extremity was measured at baseline, follow-up, and the last visit using a standardized tape. The circumference of the affected and unaffected extremities was measured in 6 places, 15 cm above the elbow, 10 cm above the elbow, at the elbow (using the antecubital crease as a reference point), 10 cm below the elbow, 15 cm below the elbow, and at the wrist. The circumference difference ratio was calculated according to the formula: (circumference of affected extremity – circumference of unaffected extremity)/circumference of unaffected extremity × 100. In addition, the volume of the extremity was calculated based on the circumference measures. The volume segment was measured according to the formula of a truncated cone: *V* = π × *h* × (*R*^2^ + *r*^2^ + *Rr*)/3, where π is a constant, *h* is the height, *R* is the radius on base, and *r* is the radius on top.^[7,8]^ The circumferences of extremities measured at 10 cm above the elbow and 10 cm below the elbow was used to calculate the volume of the segment. The volume of the unaffected extremity was also measured and the volume difference ratio was ultimately calculated according to the formula: (volume of affected extremity – volume of unaffected extremity)/volume of unaffected contralateral extremity × 100.^[9]^

### 2.2 Surgical technique and postoperative care

**Figure 2:**
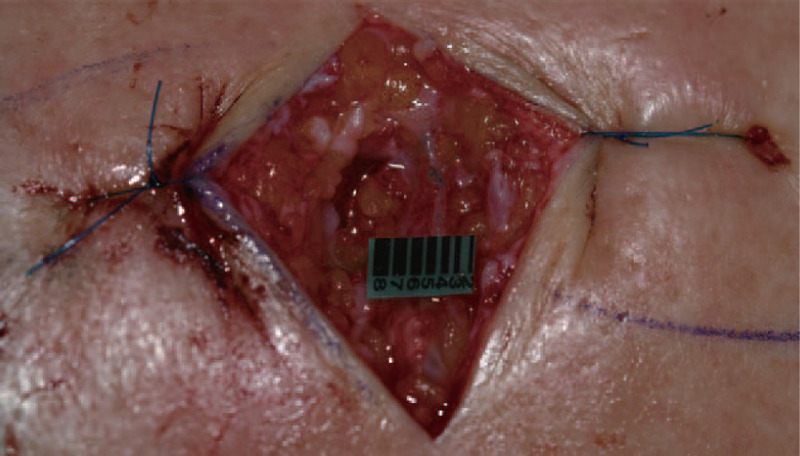
Lymphovenous anastomosis of the upper extremity. This patient underwent 4 lymphovenous anastomoses at the wrist, forearm, and anterocubital levels. An additional 1 hour of lymphovenous anastomosis may help patients relieve the symptoms of lymphedema and carpal tunnel syndrome.

Release of the transverse carpal ligament (TCL) was performed first, followed by LVA. Under general anesthesia, the patient was placed in the supine position and a tourniquet was used. Incisions were made along the palmar crease line. After making an incision in the TCL, the median nerve was identified, dissected, and the TCL was completely released. Subsequently, 4 lymphatic vessels (2 at the wrist, 1 at the medial forearm, and 1 at the antecubital fossa) distal to the areas of dermal backflow were marked for microsurgical anastomosis to adjacent small veins for LVA. Intraoperatively, 3-cm-long longitudinal incisions were made under a surgical microscope were made according to the magnetic resonance lymphangiography. After a superficial fascia incision, functioning lymphatics were identified deep in the superficial fascia and 1 to 2 functional lymphatic vessels were anastomosed to the adjacent veins using 11-0 nylon sutures (Fig. 2). Functional drainage was confirmed by the washout of venous blood in the anastomosed vein.

Antibiotics were administered to prevent cellulitis and infection postoperatively. The right arm was compressed and elevated immediately postoperatively and postoperative compression bandage therapy with 35 to 40 mm Hg pressure was instituted following surgery.

## 3 Results

**Figure 3:**
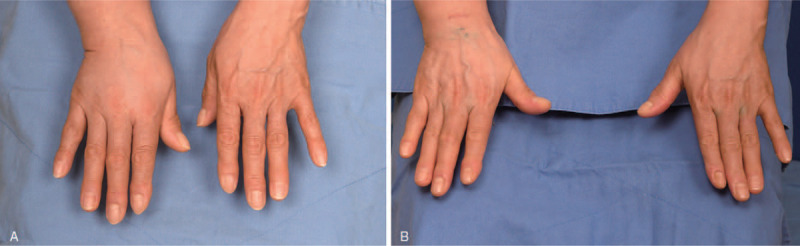
Preoperative and postoperative views of both hands (A) preoperative view. (B) Two-month postoperative view. This resulted in a significant reduction of the volume of the right hand and the patient reported improvement in the symptoms of both carpal tunnel syndrome and lymphedema.

The circumference difference ratios before surgery were 27%, 26%, 25%, 22%, 18%, and 17% 15 cm above the elbow, 10 cm above the elbow, at the elbow (antecubital crease), 10 cm below the elbow, 15 cm below the elbow, and at the wrist. The ratios were decreased to 20%, 17%, 15%, 13%, 12%, and 8% at these 6 levels, respectively. The volume difference ratio was also decreased from 51% to 31%. The patient had significant circumference and volume reduction of the right hand (Fig. 3). The CTS and lymphedema symptoms have decreased following synchronous TCL release and LVA surgeries.

## 4 Discussion

Breast cancer-related lymphedema is a chronic disease that affects 15% to 30% of breast cancer survivors.^[10]^ Previous studies related to the presence of neuropathic pain in patients with breast cancer-related lymphedema reported neuropathic pain in 30% of the patients.^[11]^ In addition, the degree of neuropathic pain in patients with breast cancer-related lymphedema was more often moderate than mild.^[12]^ In this study, symptoms related to CTS developed since lymphedema worsened 4 years after mastectomy and lymphadenectomy. Therefore, the cause of CTS may be related to the severity of the lymphedema rather than lymphadenectomy and the development of lymphedema in this case.

Selection of the proper surgical method to treat lymphedema may be important achieving a favorable result. Traditionally, LVA was considered for early-stage lymphedema but functional LVA may effectively reduce the volume of the limb and improve the subjective symptoms of patients with advanced-stage lymphedema.^[9]^ LVA may benefit patients with advanced-stage upper extremity lymphedema when functional lymphatic vessels are identified. Recently, magnetic resonance lymphangiography and supermicrosurgery techniques have become available to successfully identify functioning lymphatic vessels and achieve favorable LVA results to improve lymphedema.

This study showed that the simultaneous release of the TCL and LVA could effectively reduce the volume and improve symptoms in the hand of a patient with advanced-stage lymphedema and CTS. Regarding the influence of lymphedema as a cause of neuropathy, previous studies have shown that lymphedema was a risk factor for aggravating CTS symptoms. Ganel et al^[13]^ were the first to identify an association between neuropathies and lymphedema in breast cancer patients who underwent mastectomy and lymphadenectomy. They showed that 62% of the patients diagnosed with CTS had lymphedema. Bozentka et al^[14]^ also showed that the symptom of hand numbness was related to the degree of lymphedema. In this study, we believe that lymphedema was not the cause of CTS, but that the symptoms of CTS could be worse according to the severity of lymphedema. The lymphedema of the patient in this study was characterized as International Society of Lymphology late stage 2 with severe dermal backflow in the entire extremities including the hand in ICG lymphography. However, the patient symptoms related to CTS were relieved when lymphedema symptoms such as heaviness, hardness, and largeness improved after complete congestive therapy. Further investigations are needed to clarify the association between CTS symptoms and the severity of lymphedema.

Lymphedema presents a difficult treatment problem because the pathophysiology of the condition involves multiple factors. Therefore, the selection of the proper surgical method for upper extremity lymphedema may be important to achieving favorable results. For patients with stage 3 lymphedema, the clinical outcomes of the vascularized lymph node transfer for lymphedema may be superior to LVA. However, vascularized lymph node transfer is typically more invasive than LVA. In addition, donor site morbidity, long operation time, conspicuous scars, adhesion, and bulkiness following transfer at the wrist level are problems to overcome in vascularized lymph node transfer for lymphedema surgery. In contrast, LVA may be an alternative surgical option when functional lymphatic vessels can be identified using ICG lymphography. In addition, the time–cost necessary to complete LVA surgery was higher than that for vascularized lymph node transfer. An increase in operative time of 1 to 2 hours following the release of the TCL may improve the symptoms related to both CTS and lymphedema, consequently increasing the quality of life in CTS patients with advanced-stage lymphedema.

To the best of our knowledge, this study was the first case report to demonstrate the effectiveness of simultaneous LVA and release of the TCL in a patient with advanced-stage lymphedema and concurrent CTS. Although a further case series with large numbers of patients and a long-term follow-up period is needed, simultaneous LVA and release of the TCL may be effective and safe in patients with advanced lymphedema and CTS.

## Acknowledgments

The author is indebted to Eun-Sang Dhong, Deok-Woo Kim, and Eul-Sik Yoon who shared their experience in the microsurgical technique.

## Author contributions

**Conceptualization:** Kyung-Chul Moon.

**Data curation:** Soo-Byn Kim, Kyung-Chul Moon.

**Formal analysis:** Kyung-Chul Moon.

**Funding acquisition:** Kyung-Chul Moon.

**Investigation:** Kyung-Chul Moon.

**Methodology:** Kyung-Chul Moon.

**Project administration:** Kyung-Chul Moon.

**Resources:** Kyung-Chul Moon.

**Software:** Kyung-Chul Moon.

**Supervision:** Kyung-Chul Moon.

**Validation:** Kyung-Chul Moon.

**Visualization:** Kyung-Chul Moon.

**Writing – original draft:** Soo-Byn Kim, Kyung-Chul Moon.

**Writing – review & editing:** Kyung-Chul Moon.
